# Oxygen-Assisted B–N Codoping Enables Shallow BN_2_ Donors for n-Type Diamond

**DOI:** 10.34133/research.0994

**Published:** 2025-12-15

**Authors:** Dongliang Zhang, Xiang Sun, Wei Shen, Qijun Wang, Zhaofu Zhang, Chunmin Cheng, Yunfei Song, Jianshu Liu, Fang Dong, Zhiyin Gan, Gai Wu, Yuzheng Guo, Sheng Liu, Chingping Wong

**Affiliations:** ^1^ School of Integrated Circuits, Wuhan University, Wuhan 430072, China.; ^2^ School of Mechanical Engineering, Huazhong University of Science and Technology, Wuhan 430074, China.; ^3^ The Institute of Technological Sciences, Wuhan University, Wuhan 430072, China.; ^4^ School of Power and Mechanical Engineering, Wuhan University, Wuhan 430072, China.; ^5^ Wuhan University Shenzhen Research Institute, Shenzhen 518057, China.; ^6^ School of Materials Science and Engineering, Georgia Institute of Technology, Atlanta, GA 30332, USA.

## Abstract

Achieving stable n-type conductivity in diamond has remained a central challenge due to the deep donor levels and compensation associated with conventional dopants. Here, we show that oxygen-assisted B–N codoping during microwave plasma chemical vapor deposition activates shallow BN_2_ donors within a narrow thermal window (~1,020 K), yielding reproducible electron conduction. First-principles calculations identify the BN_2_ complex—stabilized via oxygen-mediated regulation of N site occupancy that suppresses deeper N-related defects—as a shallow and strain-tolerant donor, consistent with the experimentally optimized growth window for BN_2_ codoping. The resulting films exhibit electron concentrations above 10^19^ cm^−3^ with a mobility of 4.05 cm^2^/(V·s) and a shallow activation energy of ~21.1 meV, confirming effective n-type transport. Integration with p-type 2H-MoTe_2_ yields a diamond-based pn junction with clear rectifying behavior. While device performance is presently limited by interface states, these findings establish BN_2_ codoping as a viable strategy for n-type diamond and highlight interface quality as the decisive frontier for diamond electronics, opening opportunities for high-frequency, high-power, and radiation-hard device platforms.

## Introduction

Diamond is a material of substantial interest for a wide range of industrial applications. While recent advancements in ultrahard diamond [1–3] have been notable, these developments do not extend to semiconductor applications. Nevertheless, diamond is increasingly regarded as a promising platform for future micro/nanoelectronic and power devices, including high-frequency, high-power, and microelectromechanical systems/nanoelectromechanical systems (MEMS/NEMS)-based systems [4,5], owing to its exceptional characteristics, such as a wide bandgap (5.47 eV), a high breakdown field (~10 MV·cm^−1^), superior thermal conductivity (>2,000 W·m^−1^·K^−1^), and high intrinsic carrier mobilities (exceeding 3,200 cm^2^·V^−1^·s^−1^ for holes and in the range of 2,400 to 4,500 cm^2^·V^−1^·s^−1^ for electrons at room temperature [RT]) [6–8]. These unique properties make diamond highly attractive for integration into micro/nanoscale device architectures. While p-type diamond is readily achieved through boron (B) doping [9], the realization of n-type diamond remains a great challenge and constitutes the primary barrier to the fabrication and integration of diamond-based electronic and micro/nanoscale devices [10–12].

To date, limited progress has been made in achieving n-type doping through substitutional phosphorus incorporation via microwave plasma chemical vapor deposition (MPCVD) [13]. Despite some successes, phosphorus-doped diamond continues to exhibit suboptimal performance, as the Hall mobility and electron concentration at RT fail to attain the required values concurrently, as illustrated in Table S1. The highest reported electron mobility in phosphorus-doped diamond via microwave plasma-enhanced chemical vapor deposition (MPECVD) is 1,060 cm^2^·V^−1^·s^−1^, yet the carrier concentration remains at approximately 1 × 10^9^ cm^−3^ [14], which is 8 orders lower than that needed for practical use (>10^17^ cm^−3^, resistivity <1 Ω·cm [5,7]). Alternative dopants such as nitrogen, arsenic, sulfur, oxygen (O), and lithium have been explored, yet they consistently yield high resistivity and deep donor levels, underscoring the lack of a viable doping route [15–18].

Codoping strategies have been explored as a potential pathway to achieving n-type diamond. For instance, n-type BO codoped diamond has been synthesized using the high-pressure high-temperature (HPHT) method, yielding an electron concentration of 0.778 × 10^21^ cm^−3^. However, the electron mobility remains low, with a value of only 0.58 cm^2^·V^−1^·s^−1^ at RT [19]. Additionally, the HPHT method is not conducive to large-scale diamond production when compared to MPCVD, which is a widely adopted micro/nano fabrication approach, thereby limiting its feasibility for semiconductor and microsystem applications. Other types of n-type diamond are also summarized in Table S1.

Guided by first-principles calculations under BNO-relevant chemical potentials, we demonstrate that oxygen-assisted B–N codoping during MPCVD selectively stabilizes shallow BN_2_ donors within a narrow thermal window (~1,020 K), enabling n-type transport. By precisely tuning the growth temperature in a custom-built MPCVD system [20], we experimentally control the incorporation of B, N, and O dopants to selectively promote BN_2_ formation. Systematic structural, spectroscopic, and electrical characterizations—combined with Hall-effect measurements—unambiguously confirm the emergence of n-type conduction. Furthermore, we demonstrate a pn heterojunction by integrating the synthesized n-type diamond with p-type 2H-MoTe_2_. These findings establish oxygen-assisted BN codoping activation of shallow BN_2_ donors for n-type diamond as a viable strategy for n-type diamond and point toward its integration in high-frequency, high-power, and radiation-hard micro/nanoelectronic platforms.

## Results and Discussion

Achieving robust n-type conductivity in diamond has long been hindered by deep donor levels and compensation effects. Here, we demonstrate reproducible n-type conduction in boron–nitrogen–oxygen (BNO) codoped diamond, achieved through precise thermal control during MPCVD growth.

### Shallow donor activation in BN_2_ diamond

Hall effect measurements were performed on BN- and BNO-doped diamond films synthesized at substrate temperatures of 980, 1,020, and 1,060 K. To eliminate ambiguity stemming from contact resistance or interface effects, special attention was given to the fabrication of high-quality ohmic contacts. Ti/Pt/Au (20/30/100 nm) multilayer electrodes were deposited via electron beam evaporation and annealed following established protocols. As shown in Fig. S1, the Ti/Pt/Au contacts exhibit linear current–voltage characteristics with a resistance of ~80 Ω, confirming high-quality ohmic behavior. This validation rules out contact-related artifacts in Hall measurements and ensures that the extracted electron transport originates from intrinsic BNO donor states.

As summarized in Table 1, all BN-doped samples exhibit positive Hall coefficients, confirming p-type conduction. In sharp contrast, the BNO-doped sample grown at 1,020 K (BNO-B) exhibited a negative Hall coefficient of −0.202 cm^3^/C, corresponding to an electron concentration of 3.09 × 10^19^ cm^−3^ and an electron mobility of 4.05 cm^2^/(V·s). These values unambiguously confirm the emergence of n-type conduction under this specific codoping and thermal condition. Only around ~1,020 K does the oxygen-assisted B–N chemistry favor BN_2_ formation, yielding negative Hall coefficients (*n* ≈ 3.09×10^19^ cm^−3^, *μ* ≈ 4.05 cm^2^ V^−1^ s^−1^) and a shallow activation energy (~21.1 meV) consistent with BN₂ ionization.

**Table 1.** Sample names, growth conditions (power [*P*], pressure [*p*], temperature [*T*], and time [*t*]), and Hall effect measurements at room temperature of B–N codoped and oxygen-assisted B–N codoped diamond ([100]-oriented type IIa single-crystal) produced in this study.

Sample names	Growth conditions	Hall effect results
Sample-BN-A	*P* = 3,600 W *p* = 170 mbar; *T* = 980 K *t* = 2 h	*ρ* = 4.862 × 10^−2^ ohm·cm *R*_H_ = 2.900 × 10^−2^ cm^3^/C *p* = 2.15 × 10^20^/cm^3^ *μ*_p_ = 0.600 cm^2^/(V·s)
Sample-BN-B	*P* = 4,000 W *p* = 170 mbar; *T* = 1,020 K *t* = 2 h	*ρ* = 4.178 × 10^−2^ ohm·cm *R*_H_ = 1.710 × 10^−2^ cm^3^/C *p* = 3.660 × 10^20^/cm^3^ *μ*_p_ = 0.408 cm^2^/(V·s)
Sample-BN-C	*P* = 4,300 W *p* = 170 mbar; *T* = 1,060 K *t* = 2 h	*ρ* = 4.949 × 10^−2^ ohm·cm *R*_H_ = 7.700 × 10^−3^ cm^3^/C *p* = 8.100 × 10^20^/cm^3^ *μ*_p_ = 0.156 cm^2^/(V·s)
Sample-BNO-A	*P* = 3,600 W *p* = 170 mbar; *T* = 980 K *t* = 2 h	*ρ* = 7.718 × 10^−3^ ohm·cm *R*_H_ = 3.190 × 10^−3^ cm^3^/C *p* = 1.954 × 10^20^/cm^3^ *μ*_p_ = 4.140 cm^2^/(V·s)
Sample-BNO-B	*P* = 4,000 W *p* = 170 mbar; *T* = 1,020 K *t* = 2 h	*ρ* = 4.988 × 10^−2^ ohm·cm *R*_H_ = −2.020 × 10^−1^ cm^3^/C *n* = 3.090 × 10^19^/cm^3^ *μ*_n_ = 4.050 cm^2^/(V·s)
Sample-BNO-C	*P* = 4,300 W *p* = 170 mbar; *T* = 1,060 K *t* = 2 h	*ρ* = 4.844 × 10^−2^ ohm·cm *R*_H_ = 1.700 × 10^−2^ cm^3^/C *p* = 3.680 × 10^20^/cm^3^ *μ*_p_ = 0.350 cm^2^/(V·s)

*μ*_p_/*μ*_n_, carrier mobility; *ρ*, electrical resistivity; *p*/*n*, carrier concentration; *R*_H_, Hall coefficient

**Fig. 1.**
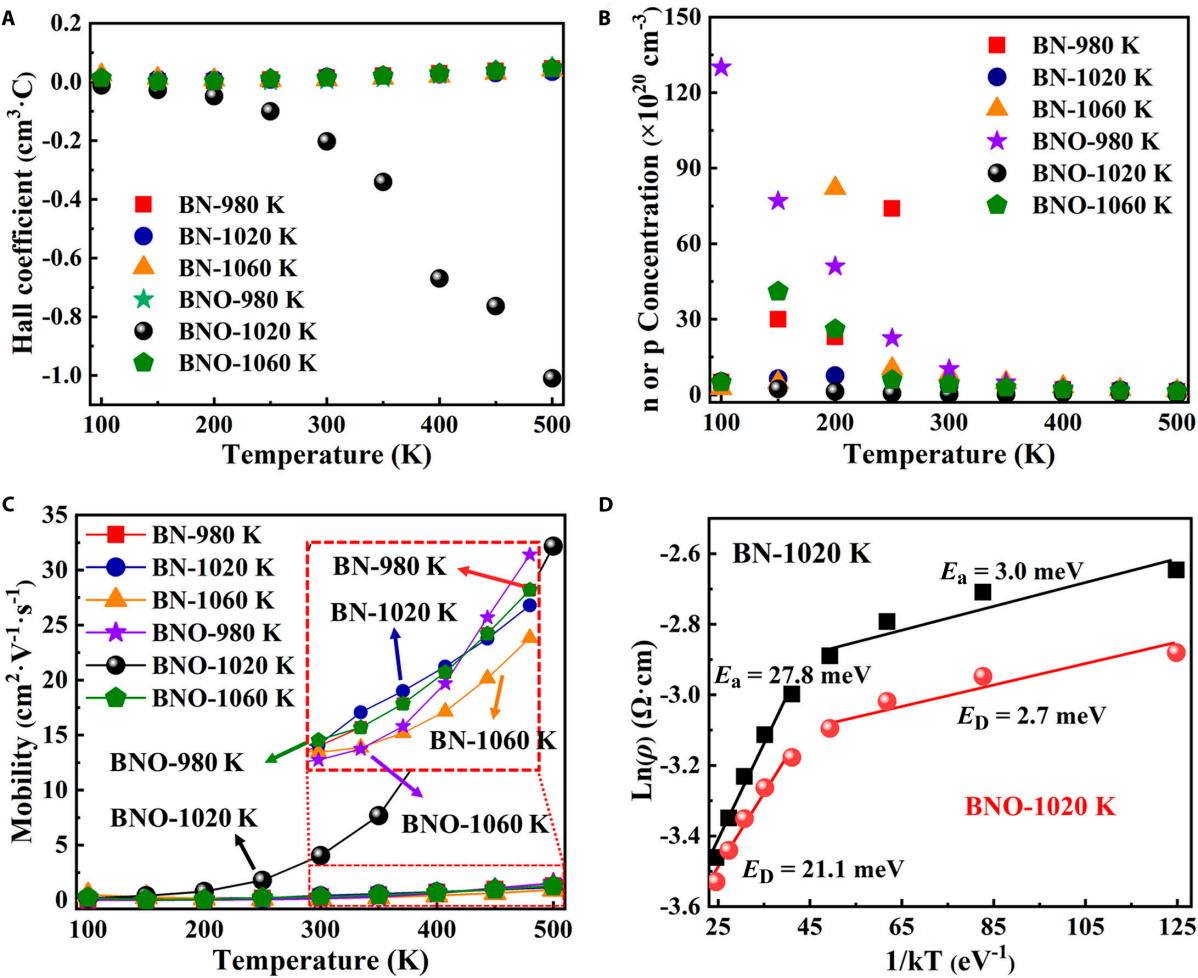
Electrical properties of codoping diamond. (A) Hall coefficient as a function of temperature. (B) Carrier concentration as a function of the reciprocal temperature. (C) Mobility as a function of temperature. (D) Log of sheet resistance of codoped diamond as a function of (kT)^−1^.

The n-type behavior is further substantiated by temperature-dependent transport measurements. As shown in Fig. 1A to C, the Hall coefficient remains negative throughout the entire 98.15 to 498.15 K range, while the conductivity increases monotonically with temperature, consistent with thermally activated donor behavior. The Arrhenius plot of sheet resistance (Fig. 1D) exhibits 2 linear regimes. At cryogenic-low T, the apparent activation energy is very small (few tens of meV), consistent with impurity-band/band-tail-assisted hopping transport that suppresses Hall voltages. At intermediate-high T, the slope yields *E*_D_ ≈ 21.1 meV, which we assign to shallow-donor ionization consistent with the proposed BN_2_ donor (~0.06 eV below CBM). This 2-regime picture (low-T hopping vs. high-T donor ionization) is widely observed in heavily doped diamond and related wide-bandgap systems [21–23].

This shallow ionization level ensures efficient donor activation at RT, making the oxygen-assisted B–N codoping scheme more compatible with low-power micro/nanoscale device integration.

The observed increase of mobility with T together with a slight decrease of Hall electron density is consistent with ionized-impurity-limited transport and Hall-extraction bias in heavily doped diamond. In the impurity-scattering regime, mobility can rise with T (Brooks-Herring/Conwell-Weisskopf), while Hall factor variations and multichannel conduction (tail/impurity-band) bias the inferred Hall electron density downward even if the ionized donor count does not decrease. Reports on doped diamond document impurity-controlled mobility branches and low-T hopping contributions.

These experimental metrics align closely with theoretical predictions for oxygen-assisted B–N codoped diamond, where adsorption energy analyses (Tables S5 to S7) reveal that O competes for nearby lattice sites, suppressing excess N incorporation and thermodynamically favoring BN_2_ formation within the observed 1,020 K window. This mechanism explains the temperature sensitivity of n-type conduction and confirms BN_2_ as the active shallow donor (~0.06 eV below CBM), consistent with the measured 21.1 meV activation energy.

Critically, the observed n-type conductivity is highly sensitive to growth temperature, underscoring that BNO incorporation can activate shallow donor complexes within a narrow thermal window, thereby overcoming deep-level and compensation limitations and enabling n-type diamond layers suitable for microsystem integration. To assess the reproducibility of this behavior, we synthesized multiple BNO-doped samples under identical conditions and subjected them to Hall measurements. As detailed in Table S2, all samples consistently exhibited negative Hall coefficients and electron conduction, providing strong experimental validation.

The observed trend—mobility increasing while the Hall electron concentration slightly decreases with temperature—can be explained by the transport regime and Hall extraction. In heavily doped wide-bandgap semiconductors like for oxygen-assisted B–N codoped diamond, the 98.15 to 498.15 K range is often ionized-impurity-limited, the effective impurity-scattering cross-section weakens with increasing temperature, and *μ*(T) can rise before phonon scattering dominates at higher T (Brooks-Herring/Conwell-Weisskopf picture). Meanwhile, a temperature-dependent Hall factor and possible multichannel conduction (tail/impurity-band contributions at low-intermediate T) bias the Hall density downward even if the total ionized-donor count does not decrease. These effects are consistent with prior analyses and temperature-dependent transport datasets in doped diamond [21–24].

The relationship between shallow donors and compensation mechanisms is crucial for understanding the behavior of charge carriers in the material, as the shallow BN_2_ donor ensures that shallow-donor activation dominates the high-T Arrhenius slope, while residual compensation and trap-assisted channels at lower T (tail/impurity-band) reduce the Hall-visible free-electron population without necessarily degrading mobility in the impurity-limited regime. This behavior differs from textbook extrinsic semiconductors but agrees with heavily doped diamond, where impurity scattering, Hall-factor variation, and hopping/impurity-band transport can coexist over adjacent T windows [22,23].

### Microscale uniformity and defect control

To establish a direct link between lattice quality and electronic performance, a set of micro/nanoscale structural and spectroscopic characterizations was conducted. Such analyses are critical for ensuring material reliability in micro/nanoelectronic device contexts.

**Fig. 2.**
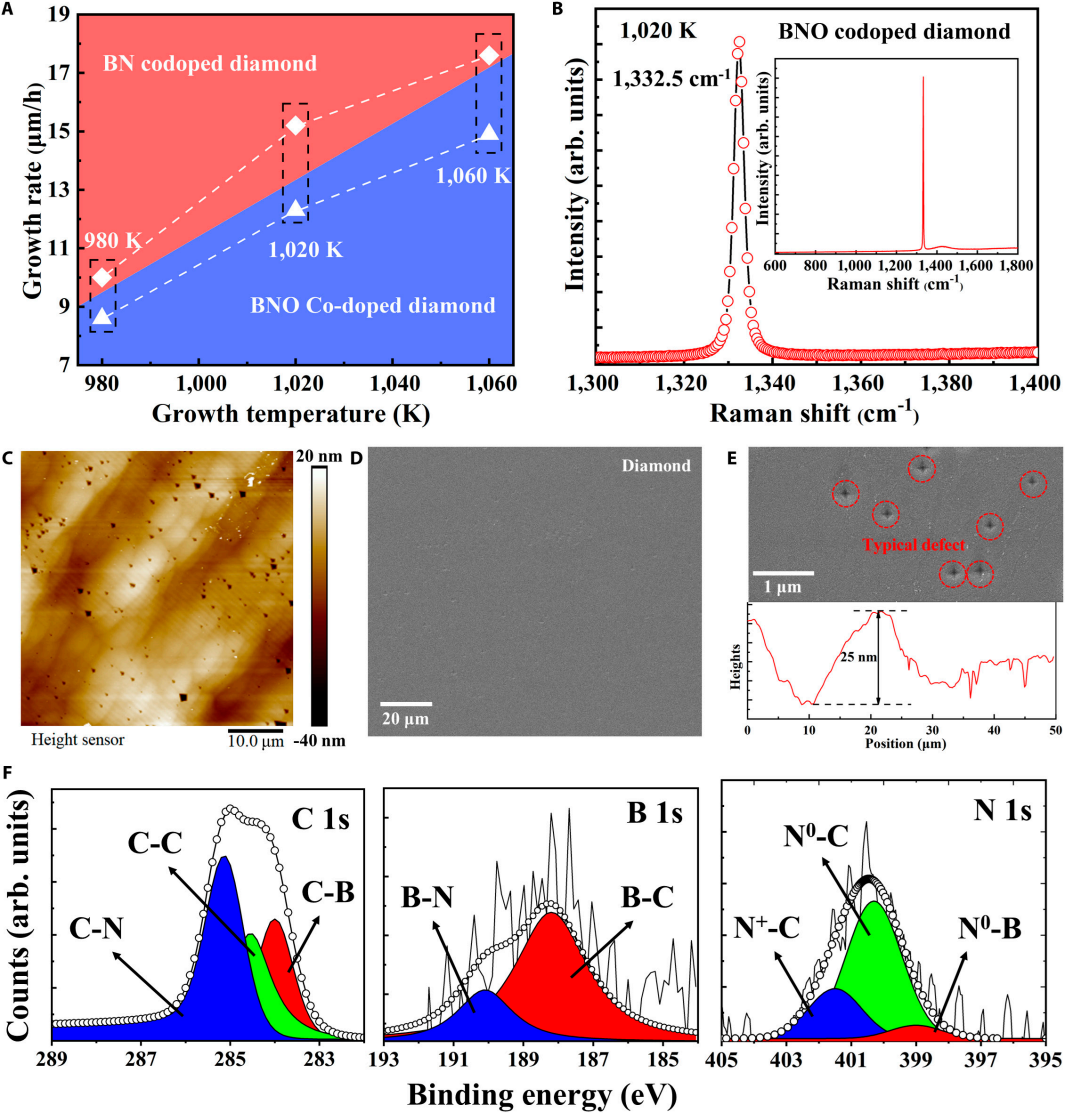
Growth characteristics, surface morphology, and chemical state analysis of codoped diamond films. (A) Growth rate of codoped diamond as a function of temperature. (B) Raman spectrum of BNO codoped diamond synthesized at 1,020 K. (C) AFM image. (D) SEM image. (E) Typical defect and (F) XPS spectra of C 1s, B 1s, and O 1s of BNO codoped diamond synthesized at 1,020 K.

Figure 2A illustrates the temperature-dependent growth rates of BN and BNO codoped diamond films, highlighting the optimal rate for BNO doping. Raman spectroscopy shows a sharp 1,332.5 cm^−1^ peak with reduced FWHM and stress, evidencing defect suppression relative to singly doped films, shown in Fig. 2B. Raman mapping (Fig. S2) further confirms spatially uniform stress from center to edge, establishing microscale uniformity as a prerequisite for reproducible device stability.

Atomic force microscopy (AFM) (Fig. 2C) and scanning electron microscopy (SEM) (Fig. 2D) reveal dense step-flow epitaxy with low roughness and minimal topographical defects (Fig. 2E). In our MPCVD growth on {100} substrates, the “typical defects” (Fig. 2C and E) arise from step-flow growth on {100} where local surface reconstruction leads to the exposure of {111} facets. The pyramidal hillock is consistent with step bunching and facet-selective kinetics (N promoting {111}, O tuning surface bonding), which locally increase the {111} growth rate and stabilize faceted pyramids. Conversely, the truncated inverse pyramid/etch-pit in Fig. 2E can nucleate at step terminations or dislocation outcrops under transient supersaturation (radical-flux/pressure) variations, leaving {111}-bounded pits.

Consistent with an oxygen-assisted mechanism, the BNO samples show no reproducible, fit-quality O 1s in 529 to 535 eV (below ~0.1 to 0.2 at.% detection), supporting the view that oxygen primarily regulates the defect landscape rather than functioning as a lattice donor. Cross-checks (C 1s/B 1s/N 1s) likewise show no robust O-related shoulders. X-ray photoelectron spectroscopy (XPS, Fig. 2F) provided further chemical evidence of dopant incorporation. The C 1s spectrum exhibits a dominant peak at 284.8 eV (sp^3^ C–C bonding), with shoulder features at 283.5 and 285.2 eV corresponding to C–B and C–N bonds. The B 1s spectrum shows components at 188.0 eV (B–C) and 190.1 eV (B–N), while the N 1s spectrum reveals peaks at 398.5 eV (N–B), 399.7 eV (neutral substitutional N^0^–C), and 401.3 eV (positively charged N^+^–C). The coexistence of these chemically distinct bonding states demonstrates stable dopant incorporation at the nanoscale, which is essential for long-term heterojunction reliability in integrated micro/nanoelectronic systems.

The XPS of oxygen-assisted B–N codoped diamond shows no reproducible, fit-quality O 1s component in 529 to 535 eV, consistent with oxygen below the practical XPS detection limit (~0.1 to 0.2 at.%) under our acquisition conditions. To suppress adventitious adsorbates, we performed 3 “in situ gentle Ar^+^ sputter, then immediate O 1s acquisition” cycles before each scan; O 1s remained nondetectable, reinforcing that bulk/near-surface O is below detectability. Notably, O 1s does not overlap with C 1s (~284 to 290 eV); the perceived “overlap” originates within the O 1s envelope itself where the high-BE side (~533 to 534 eV) can coincide with adsorbed H_2_O/COx O 1s and mask very weak lattice-related signals. Cross-line checks likewise show no O-consistent shoulders in C 1s (C–O 286.3 to 286.7 eV; C=O 287.8 to 288.2 eV), B 1s (B–O 192 to 193 eV), or N 1s (N–O ~402 eV), and energy dispersive x-ray spectroscopy (EDS)—less sensitive to light elements—also reports no O, all consistent with an ultralow O content. Mechanistically, under MPCVD conditions, oxygen most plausibly acts as a kinetic/defect modifier—suppressing deep N complexes and promoting the shallow BN_2_ donor—without forming abundant bulk B–O/C–O bonds resolvable by surface-sensitive XPS.

Collectively, these nanoscale-resolved analyses confirm that the observed n-type conduction originates from well-incorporated oxygen-assisted B–N codoped diamond while preserving lattice integrity. Raman mapping further verified spatially uniform stress across center and edge regions (Fig. S3), and AFM profiles revealed sub-nanometer rms roughness, both highlighting microscale homogeneity essential for reproducible device performance, thereby providing a structural foundation for the theoretical donor configuration analysis discussed in the next section.

### BN_2_ complexes as stable shallow donors

**Fig. 3.**
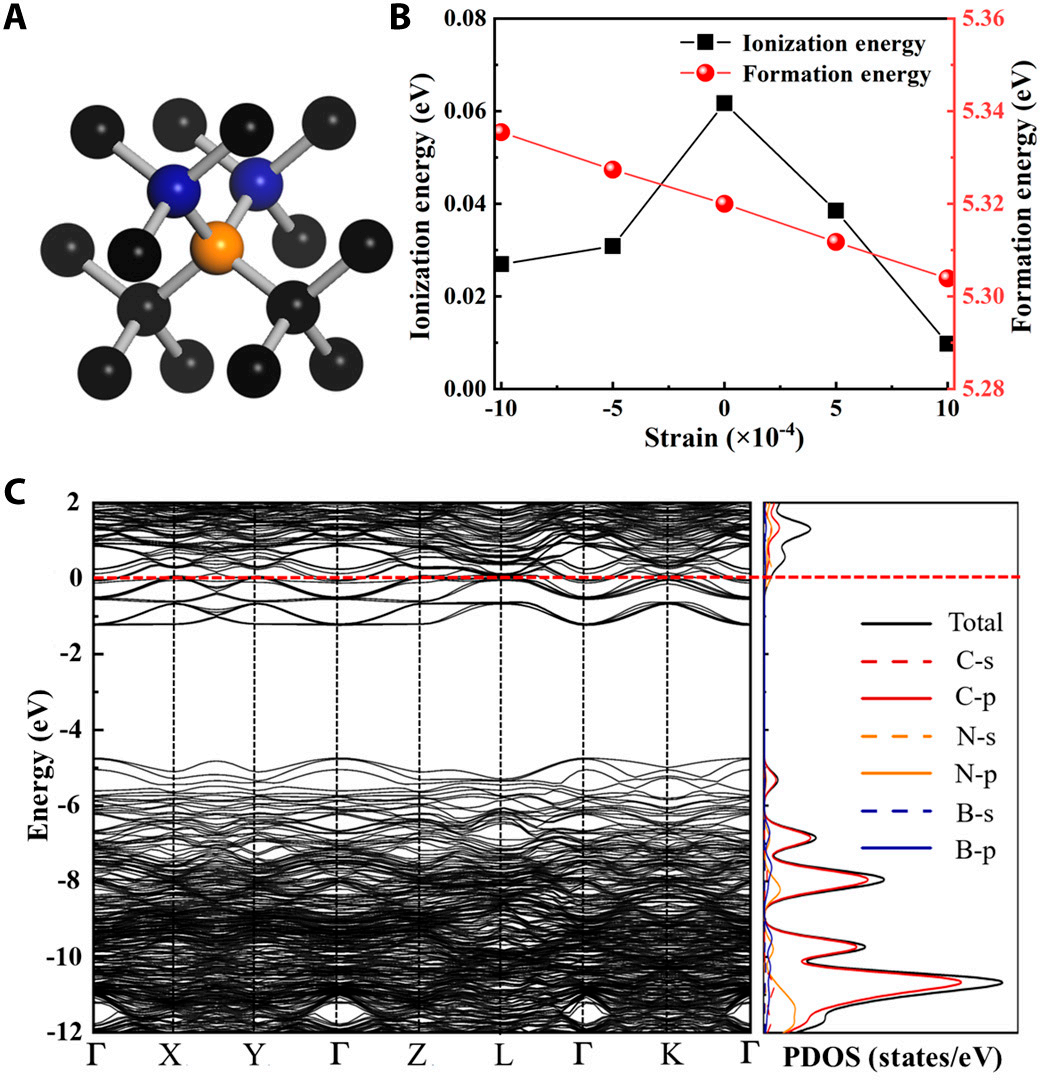
Theoretical results of BN_2_ impurity states in diamond. (A) Optimized structures. (B) Variation of formation and ionisation energies with strain. (C) The calculated band structures and PDOS for BN_2_-diamond.

First-principles calculations (details in the Supplementary Materials) were carried out to identify the donor states responsible for the observed n-type conduction in oxygen-assisted B–N codoped diamond. Among the considered defect complexes (BN, BN_2_, BO_3_, BO_4_, etc.), the BN_2_ configuration emerged as the most promising shallow donor, with a formation energy of 5.32 eV and a donor level only ~0.06 eV below the conduction band minimum (Fig. 3C and Table S3). Although this formation energy appears high compared with conventional dopants, it is substantially lowered under N-rich and high-temperature MPCVD conditions, where nitrogen chemical potential shifts make BN_2_ incorporation thermodynamically favorable. This explains why BN_2_ emerges selectively at ~1,020 K, fully consistent with our experimental observations. The optimized BN_2_ geometry retains near-ideal diamond bond lengths (~1.54 Å), ensuring minimal lattice distortion and compatibility with micro/nanoscale device integration. Our analyses underscore the BNO synergy: B–N hybridization yields conductive states near the CBM, refined by O’s defect suppression, which optimizes charge transfer and aligns with the experimentally validated n-type transport characteristics. Under BNO-relevant chemical potentials, oxygen competes for N-favorable sites, suppressing deep N complexes and lowering the effective barrier toward BN_2_ stabilization; this oxygen-assisted pathway quantitatively explains the experimental window (~1,020 K) and the shallow activation (~21.1 meV).

Mechanical robustness is particularly important for diamond-based devices operating under thermal and stress conditions. Calculations under biaxial strain confirmed that BN_2_ remains electronically active and stable, unlike other donor configurations that become deep or unstable (Fig. 3B). This strain tolerance suggests that BN_2_ donors can maintain functionality in micro/nanoelectronic platforms such as MEMS/NEMS resonators and high-power transistors.

Hydrogen passivation, a known deactivation route in MPCVD-grown diamond, was also examined. Our calculations show that hydrogen incorporation at BN_2_ sites is energetically unfavorable (Table S4), implying that BN₂ donors are less prone to passivation than conventional dopants. This feature further enhances their reliability for device-level applications.

Adsorption-energy analyses (Tables S5 to S7 and Fig. S4) show that boron facilitates nitrogen incorporation while oxygen competes for nearby sites, creating a narrow thermal window (about 1,020 to 1,030 K) in which BN_2_ complexes are preferentially stabilized and can act as shallow donors. Specifically, under B_2_H_6_/N_2_/O_2_ conditions mimicking MPCVD, O’s higher binding affinity at interstitial N sites acts as a site blocker, preventing the formation of deeper donor complexes like N–C or N–N pairs that would otherwise compensate or deepen the donor levels. This competitive mechanism lowers the effective formation energy of BN_2_ from ~5.32 eV (in isolation) to lower energy, rendering it the most stable configuration in the full BNO system. This O-mediated regulation of N defect structures is experimentally corroborated in synthetic diamonds, where O incorporation visibly alters the color of N-doped diamonds (e.g., from yellow to colorless), reflecting the macroscopic effect of O modifying defect electronic structures via competitive site occupation, in line with our theoretical model. Differential charge density analysis (Fig. S4) further illustrates how this O-mediated stabilization localizes electron density near the BN_2_ site, facilitating the observed shallow donor behavior (0.06 eV below CBM) and reproducing the experimental electron concentrations (>10^19^ cm^−3^) and low activation energies (~21 meV). Notably, this prediction matches experimental observations: only samples grown at 1,020 K displayed reproducible n-type conduction (Fig. 1 and Table S2). These results establish a direct correlation between thermodynamic driving forces and donor activation, highlighting how growth-condition optimization translates into electronic performance relevant for micro/nanoelectronic device fabrication.

Compared with conventional n-type routes (P or S), the BNO strategy shows superior prospects for long-term stability and passivation resistance. Experimentally, the Hall coefficient remains negative across 98 to 498 K, and Arrhenius analysis of the sheet resistance gives a shallow activation energy of ~21.1 meV, securing robust donor activation within device-relevant temperatures. In prior reports, P/S-doped diamonds often face deep donor levels and/or unfavorable carrier density-mobility trade-offs (see Table S1). First-principles calculations further indicate that hydrogen incorporation at BN_2_ sites is thermodynamically unfavorable across tested positions, implying intrinsic tolerance against H-related passivation. Together with the strain-tolerant shallow level of BN_2_, these results support the long-term stability advantage of the BNO route.

### Pn heterojunction and interface challenges

**Fig. 4.**
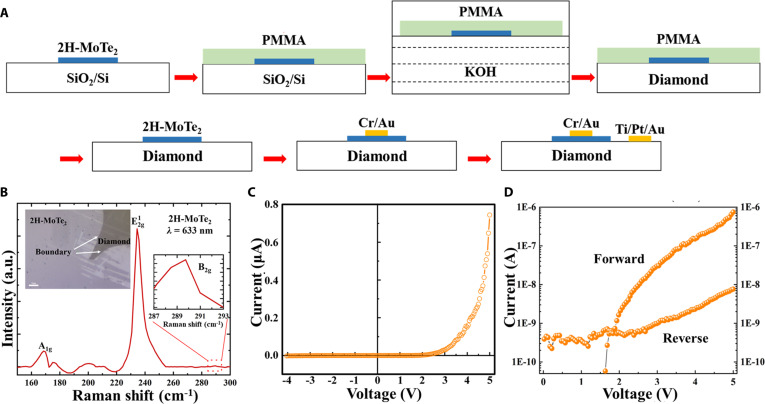
Diamond-based pn junctions with p-type 2H-MoTe_2_ composition. (A) Fabrication process of the heterojunction pn junction. (B) Raman spectra of 2H-MoTe_2_ and diamond with excitation at a wavelength of 633 nm. Current–voltage (*I*–*V*) characterization of the diamond and 2H-MoTe_2_ pn junctions: (C) linear plot and (D) semilogarithmic plot.

To support the successful synthesis of oxygen-assisted B–N codoped n-type diamond, we constructed a pn heterojunction demonstration with p-type 2H-MoTe_2_ [25], shown in Fig. 4A. This contrasts with the performance limitations of early P-doped homo-junctions [26], highlighting the potential of our n-type approach. Future p-n homo-junctions and deep UV luminescence testing will further expand its optoelectronic applications.

In this study, the 2H-MoTe_2_ used for the PNJ is approximately 5 layers thick, with a total thickness of around 3.25 nm. The diamond layer for the n-type layer in the junction is about 20 μm thick. Patterned transfer and verified ohmic contacts on diamond ensured reliable junction probing. This demonstration serves as a critical bridge from material-level validation to micro/nanoelectronic device applications.

Ohmic contacts on the diamond side were defined with Ti/Pt/Au multilayers, yielding linear *I*–*V* characteristics (Fig. S1), ensuring accurate probing of the n-type diamond channel. On the MoTe_2_ side, Cr/Au electrodes were employed to ensure reliable p-type injection. The p-type nature of 2H-MoTe_2_ was further confirmed by field-effect transistor (FET) transfer measurements (Fig. S5), which displayed unipolar hole transport. The transferred 2H-MoTe_2_ flake used in the pn junction is ~5 layers, consistent with the p-type transfer characteristics in Fig. S5. This independent verification ensures that the heterojunction polarity arises from intrinsic conduction rather than extrinsic effects.

These validations confirm the well-defined polarity of both diamond and MoTe_2_, ensuring that the measured heterojunction response originates from intrinsic pn conduction.

The structural integrity of 2H-MoTe_2_ after transfer was confirmed by Raman spectroscopy, which displayed the characteristic peaks at 169.3, 234.8, and 289.9 cm^−1^ without detectable broadening or shifts (Fig. 4B). The Raman spectra shown in the manuscript confirm the bandgap and crystal quality of p-type MoTe_2_, which are essential for its role in electronic and optoelectronic applications. This microscale verification rules out structural degradation or phase transformation, confirming that the heterojunction retains high crystalline quality.

The pn junction exhibits clear rectification with a turn-on voltage of ~2 V and a rectification ratio of ~100 at ±5 V, confirming functional pn conduction, shown in Fig. 4C. The *I*–*V* characteristics of the pn junction are also presented in a semilogarithmic scale to clearly demonstrate the rectifying behavior, as shown in Fig. 4D. This semilogarithmic representation highlights the current rectification at both forward and reverse biases, with reverse leakage current and the on/off ratio becoming more apparent.

The modest rectification ratio observed in the pn junction can be attributed to several interface-related issues. The interface states, arising from the lattice mismatch between diamond and MoTe_2_, can act as traps for the charge carriers, leading to reduced carrier injection efficiency. These interface traps contribute to the nonideal diode behavior, resulting in a lower rectification ratio than expected for an ideal pn junction. Additionally, the barrier inhomogeneity at the interface between the 2 materials can lead to local variations in the band alignment, which further impacts the current conduction.

From a broader perspective, the reproducible demonstration of a functional pn heterojunction confirms that n-type diamond can be reliably integrated into hybrid device architectures. Despite current interface limitations, this result underscores the transformative potential of diamond–2-dimensional (2D) heterostructures for high-frequency rectifiers, low-leakage photodetectors, and radiation-hardened circuits within micro/nanoscale systems.

In summary, while current performance is interface-limited, the demonstration of a functional pn heterojunction confirms device-level viability and highlights interface engineering as the decisive frontier to unlock n-type diamond for high-frequency, high-power, and radiation-hard micro/nanoelectronics.

Optimized doping concentrations ([B]/[C] = 625 parts per million [ppm], [N]/[C] = [O]/[C] = 25,000 ppm) are critical for high-power applications, enabling high carrier concentration and low resistivity while minimizing compensation traps. Theoretical analysis from density functional theory (DFT) shows that higher N/O levels (>30,000 ppm) increase formation energies of deep defects, reducing *n* and impairing mobility under thermal stress, whereas balanced concentrations stabilize shallow BN_2_ donors, enhancing high-power performance (e.g., >10 MV/cm breakdown tolerance). Elevated-temperature Hall measurements (Fig. 1, 98 to 498 K) validate this: *n* remains largely stable, confirming thermal robustness and simulating operational heat loads. Long-term stability under high-power conditions requires future testing to monitor sustained *n*/*μ* over extended cycles, but current reproducibility (Table S2, <10% variation) suggests excellent potential for radiation-hard devices.

Notably, elevated-temperature Hall data up to 498 K corroborate stable n-type transport across batches and—together with the calculated unlikelihood of H-passivation at BN₂ sites—suggest that oxygen-assisted B–N codoped n-type diamond is better positioned than P/S-doped counterparts to maintain electron activity under hydrogen-rich processing and service conditions.

The stabilization of the BN_2_ donor in oxygen-assisted B–N codoped n-type diamond arises within a narrow thermal window near ~1,020 K, driven by oxygen’s competition for nitrogen-adjacent lattice sites. Accordingly, scale-up prioritizes wafer-level temperature uniformity to keep all regions inside this window. Our MPCVD tool provides independent control of power/pressure/temperature with dual-wavelength pyrometry (±1 K), and center-to-edge Raman mapping already confirms microscale stress uniformity on device coupons. For large-area substrates, we anticipate (a) enforcing ±1 to 2 K uniformity via multizone heating with closed-loop pyrometry, (b) minimizing radial variations in B/N/O arrival rates by substrate rotation and flow-field optimization, and (c) feed-ratio calibration of B_2_H_6_/N_2_/O_2_ to maintain local chemistry within the BN_2_ regime. We will validate uniformity through wafer-scale Raman grids, spatially resolved XPS/EDS, and secondary ion mass spectrometry (SIMS) line-scans. These steps provide a practical engineering path to translate the lab-scale process to large-area devices.

## Conclusion

We demonstrate n-type diamond by oxygen-assisted B–N codoping that activates shallow BN_2_ donors within a narrow growth window. The role of oxygen is mechanistic (defect/kinetic regulation) rather than compositional (lattice donor), reconciling theory and experiment without implying substantial O incorporation. Guided by first-principles calculations, the BN₂ complex—enabled by O’s competitive suppression of deeper N defects, with a formation energy of 5.32 eV and donor level ~0.06 eV below the conduction band minimum—is identified as a shallow, strain-tolerant donor stabilized within a narrow growth-temperature window of oxygen-assisted B–N codoping. Experiments confirm electron concentrations of 3.090 × 10^19^ cm^−3^ and a mobility of 4.050 cm^2^·V^−1^·s^−1^ with a shallow activation energy of ~21.1 meV, validating the predicted donor mechanism. Furthermore, integration into a pn heterojunction with a 2D semiconductor illustrates device-level feasibility.

Beyond this proof-of-concept demonstration, the findings highlight shallow donor activation and interface quality as decisive factors for advancing diamond electronics. Optimized doping concentrations achieve high carrier levels essential for power devices (e.g., high-voltage FETs >10 MV/cm breakdown) and radiation detectors (>1 Mrad tolerance), while elevated-temperature Hall data (up to 498 K) confirm thermal stability under operational loads (>200 W/cm^2^ dissipation). Interface optimization is key to overcoming current limitations in pn heterojunctions (e.g., modest rectification due to trap states at diamond-MoTe_2_ boundaries); strategies such as H-termination passivation or undoped diamond buffer layers can enhance stability and ideality, as demonstrated in diamond/2D integrations and wide-bandgap heterojunctions. Future efforts in defect control and heterointerface engineering will be essential to fully unlock n-type diamond for high-frequency, high-power, and radiation-hard micro/nanoelectronic technologies.

## Methods

### Experimental details

#### Diamond film synthesis

Single-crystal diamond films were epitaxially grown on high-purity [100]-oriented type IIa single-crystal diamond substrates (Element Six Ltd., UK; dimensions: 3 × 3 × 0.5 mm^3^; nitrogen impurity level <1 ppm). Prior to growth, substrates underwent ultrasonic cleaning in acetone and isopropyl alcohol (10 min each), followed by boiling in a 1:1:1 sulfuric acid:nitric acid:perchloric acid mixture at 200 °C for 30 min to remove contaminants and graphitic residues. Substrates were then rinsed with deionized water (18.2 MΩ·cm resistivity) and dried under nitrogen flow. Nucleation was promoted via 30-min plasma pretreatment in the MPCVD chamber using 2% CH_4_/H_2_ plasma at 800 °C and 100 mbar.

Synthesis occurred in a custom-built MPCVD reactor (in-house, 6 kW/2.45 GHz magnetron) with independent control of power, pressure, and temperature (dual-wavelength pyrometry, ±1 K accuracy). Precursor gases included CH_4_ (6N, 8 sccm) as carbon source and H_2_ (7N, 200 sccm) as diluent. Dopants were introduced via B_2_H_6_ (6N, 5% in H_2_, 0.1 sccm; [B]/[C] = 625 ppm), N_2_ (6N, 0.2 sccm; [N]/[C] = 25,000 ppm), and O₂ (6N, 0.2 sccm; [O]/[C] = 25,000 ppm), optimized per simulations for BNO complex formation. Growth proceeded at 980 to 1,060 K for 2 h under continuous plasma.

#### Structural and chemical characterization

Raman spectroscopy employed a high-sensitivity Horiba LabRAM HR Evolution spectrometer (532 nm excitation, resolution <1 cm^−1^, −70 °C semiconductor-cooled CCD detector) to determine bonding types, phase purity, and stress distribution; mapping covered 500 × 500 μm^2^ areas.

Microstructure was examined via high-resolution SEM (Hitachi SU8230; 1.5 nm resolution at 10 kV) with EDAX EDS and EBSD for concurrent morphology, elemental composition, and crystallographic analysis. Surface roughness was quantified using AFM (Bruker Dimension Icon; 0.2 nm lateral resolution, 55 × 55 μm max scan, temperature-controlled stage to 300 °C) in tapping mode (5 × 5 μm^2^ areas).

Chemical states were probed by XPS (Kratos AXIS SUPRA+; Al/Ag dual anodes, ≤0.45 eV energy resolution, cluster ion gun; snapshot spectroscopy and chemical state imaging with <1 μm spatial resolution; calibrated to C 1s at 284.8 eV using CasaXPS fitting). Photoluminescence spectra (77 K, 325 nm He-Cd laser excitation; Jobin Yvon Fluorolog-3) detected NV centers and other defects, characterizing their emission and distribution.

#### Electrical characterization

Carrier transport and device properties were evaluated using an integrated platform featuring the Keysight B1500A semiconductor parameter analyzer (current/voltage resolutions: 10 fA/0.5 μV; output: ±200 V/±1 A; 4 independent source measure units (SMUs) for multiterminal testing; DC/pulsed *I*–*V*, *C*–*V* from 1 kHz to 5 MHz with ±0.1% accuracy @1 kHz/100 nF; quasi-static *C*–*V*/*C*–*t* modes) and a Hall-effect system (van der Pauw configuration, 0.5 T permanent magnet with ±0.1% uniformity; resistivity: 10^−4^ to 10^11^ Ω·sq; concentration: 10^6^ to 10^21^ cm^−3^; mobility: 1 to 10^7^ cm^2^/V·s; 1 pA current detection; variable-temperature stage: −196 to 600 °C).

For Hall measurements, 4 ohmic contact electrodes (Ti/Pt/Au: 20/30/100 nm) were formed via e-beam evaporation in square van der Pauw geometry (2 mm spacing) on the epilayer, followed by 600 °C/5 min rapid thermal annealing in N_2_ (80 Ω resistance; verified by linear *I*–*V*; Fig. S1). Transport properties (room- and temperature-dependent) were acquired from 98 to 498 K (10 K increments, ~10^−6^ mbar vacuum) using B1500A SMUs for current sourcing (1 to 10 μA) and voltage sensing, with Hall voltage under 0.5 T field. Carrier type/concentration (*n*/*p*), mobility (*μ*), and resistivity (*ρ*) were derived from Hall coefficient (*R*_H_) via standard van der Pauw analysis (error <5% from replicates); Arrhenius plots extracted activation energies.

### DFT details

The structure and electronic properties of B/N/O codoped diamond are investigated using the first-principles calculations, as implemented in the plane-wave package PWmat [27]. Geometry optimization and property calculations are performed using the PBE0 hybrid functional, based on the generalized gradient approximation of Perdew–Burke–Ernzerhof (PBE) [28] and Optimized Norm-Conserving Vanderbilt pseudopotentials [29]. A 3 × 3 × 3 periodic supercell of 216 atoms is used with plane-wave basis and periodic boundary conditions. A 2 × 2 × 2 mesh of K-point [30] is adopted. The tested energy cutoff of 70 Ry is selected during the calculation. The criterion of maximum atom forces is set as 0.01 eV/Å after all calculations. The lattice constant of diamond is calculated to be 3.566 Å, while the experimental value is 3.567 Å [31]. The lattice constant of less than 1% error can make the calculation results more reliable with the selected parameters. The calculated bandgap of diamond is about 4.19 eV, and the underestimated bandgap is due to limitations of the PBE functional.

The formation energy of different charges state is calculated as [32]:

$$\begin{array}{c} H\left(\alpha ,\;q\right)=\left\{E\left(\alpha ,q\right)+E_{\text{corr}}\left(\alpha ,\;q\right)\right\}\\ -E\left(\text{host}\right)-\sum_{i}n_{i}\mu_{i}+q\left(\varepsilon_{F}+E_{V}+∆v\right) \end{array}$$ (1)

where $E\left(\alpha ,q\right)$ represents the total energy of diamond supercell doped with defect α with the charge state of *q*; $E\left(\text{host}\right)$ represents the total energy of nondoped diamond. $n_{i}$ atoms are added (*n_i_* > 0) to or removed (*n_i_* < 0) from the diamond, and $\mu_{i}$ is the chemical potential of the atom $n_{i}$. The chemical potential of H, O, B, and N is calculated from the gas phase of H_2_, O_2_, crystalline elemental boron, and N_2_, respectively. $\mu_{C}$ varies with strain and is taken to be the total energy per atom of a 216-atom supercell of strained pure diamond. $\varepsilon_{F}$ represents the Fermi energy referenced to the valence band maximum $E_{V}$ in the bulk diamond. $E_{\text{corr}}\left(\alpha ,\;q\right)$ is an image charge interaction term, while q∆v represents a potential alignment ∆v term. The image charge interaction term, *E*_corr_ (*α,q*), is handled by the C-AP method proposed by Suo et al [32].

The charge transition level $\varepsilon_{\alpha }\left(q/q^{\prime }\right)$ for the defect α is the Fermi-level position for which the $H\left(\alpha ,\;q\right)$ is equal to $H\left(\alpha ,\;q^{\prime }\right)$ [33]:

$$\varepsilon_{\alpha }\left(q/q^{\prime }\right)=\left[H\left(\alpha ,\;q\right)-H\left(\alpha ,\;q^{\prime }\right)\right]/\left(q^{\prime }-q\right)-E_{V}-∆v$$ (2)

Ionization energy of n-type diamond is expressed as $E_{\text{ionization}}=E_{\text{bandgap}}-\varepsilon_{\alpha }\left(0/+1\right)$.

Adsorption energy analyses employ a (111) diamond slab model with a vacuum spacing of 15 Å to simulate surface doping processes.

## Data Availability

The data supporting the findings of this study are available within the article and its Supplementary Materials. Additional data are available from the corresponding authors upon reasonable request.
