# Supplementary material for: Oxygen-Assisted B–N Codoping Enables Shallow BN2 Donors for n-Type Diamond
Source: Research (Wash D C). 2025 Dec 15;8:0994. doi: 10.34133/research.0994 (PMC12703017; doi:10.34133/research.0994)
Supplement: Supplementary 1 — Figs. S1 to S5 Tables S1 to S7 [file research.0994.f1.docx]

**Supplementary Materials**

**Oxygen-assisted B-N co-doping enables shallow BN_2_ donors for n-type diamond**

Dongliang Zhang ^b, #^, Xiang Sun ^a, c, #^, Wei Shen ^a, c,d^, Qijun Wang ^a, c,d^, Zhaofu Zhang ^a, c,d^, Chunmin Cheng ^a^, Yunfei Song ^a^, Jianshu Liu ^a^, Fang Dong ^a, c, d,^, Zhiyin Gan ^b^, Gai Wu ^a, c,d, *^, Yuzheng Guo ^a, c, *^, Sheng Liu ^a, b, c,d, *^, Chingping Wong ^e^

^a^ School of Integrated Circuits, Wuhan University, Wuhan 430072, China

^b^ School of Mechanical Engineering, Huazhong University of Science and Technology, Wuhan 430074, China

^c^ The Institute of Technological Sciences, Wuhan University, Wuhan 430072, China

^d^ School of Power and Mechanical Engineering, Wuhan University, Wuhan 430072, China

^e^ School of Materials Science and Engineering, Georgia Institute of Technology, Atlanta 30332, United States

Table S1. Previous representative works on n-type diamond.

| **Year** | **Dopant** | **Method** | **Carrier Concentration (cm^-3^)** | **Carrier Mobility (cm^2^/V·s)** | **Resistivity (Ω·cm)** | **Temperature** |
| --- | --- | --- | --- | --- | --- | --- |
| 1997 | P [1] | MPCVD | ~5.5×10^15^ | 23 | ~40 | 500K |
| 1999 | S [2] | MPCVD | 1.4×10^13^ | 597 | - | Room temperature |
| 2000 | P [3] | MPCVD | ~10^11^ | 240 | - | Room temperature |
| 2003 | Boron-Deuterium  [4] | MPCVD | ~7×10^16^ | 180 | 2 | 300 K |
| 2004 | P [5] | MPCVD | 5×10^10^ | 660 | 2×10^5^ | Room temperature |
| 2005 | P [6] | MPCVD | ~10^11^ | ~350 | - | Room temperature |
| 2007 | P [7] | MPCVD | ~3×10^9^ | ~570 | - | Room temperature |
| 2011 | P [8] | Ion Implantation | 3.99×10^15^ | 143 | 10.92 | Room temperature |
| 2014 | S [9] | HFCVD | 5.43×10^17^ | 11 | 0.55-1.04 | Room temperature |
| 2016 | P [10] | PECVD | ~10^10^ | 1060 | - | 300K |
|  |  |  | ~2×10^6^ | 1500 | - | 225K |
| 2017 | O [11] | Ion Implantation | 0.386 | 126 | - | Room temperature |
| 2017 | B-S co-dopants [12] | HTHP | ~1.065×10^10^ | 628.7 | 0.993×10^6^ | Room temperature |
| 2019 | B-O co-dopants [13] | HTHP | 0.778×10^21^ | 0.58 | 0.022 | Room temperature |
| 2021 | P [14] | MPCVD | 1×10^9^ | 1060 | - | Room temperature |
| 2021 | P [15] | MPCVD | ~10^13^ | 355 | ~2×10^3^ | 450 K |
| 2021 | B-N co-dopants [16] | MPCVD | ~10^11^ | ~90 | ~2×10^5^ | 773 K |
| 2022 | Ta [17] | HFCVD, Ion Implantation | - | 522 | 0.22 | Room temperature |

Table S2. Room-temperature Hall-effect characteristics of reproducibly synthesized and characterized B-N-O co-doped [100]-oriented type IIa single-crystal diamond.

| Sample names | Hall effect results |
| --- | --- |
| 0816-S1 | *ρ* = 2.44×10^-1^ ohm·cm; *R*_H_ = -3.28×10^1^ cm^3^/C  *n* = 1.90×10^17^ /cm^3^; *μ*_n_ = 1.34×10^2^ cm^2^/(V·s) |
| 0818-S2 | *ρ* = 2.24×10^-2^ ohm·cm; *R*_H_ = -7.55×10^-3^ cm^3^/C  *n* = 8.27×10^20^ /cm^3^; *μ*_n_ = 0.34 cm^2^/(V·s) |
| 0912-S3 | *ρ* = 1.59×10^-2^ ohm·cm; *R*_H_ = -1.55×10^-2^ cm^3^/C  *n* = 4.03×10^19^ /cm^3^; *μ*_n_ = 9.77 cm^2^/(V·s) |
| 0924-S4 | *ρ* = 5.01×10^-2^ ohm·cm; *R*_H_ = -6.14×10^-2^ cm^3^/C  *n* = 1.02×10^19^ /cm^3^; *μ*_n_ = 1.23×10^1^ cm^2^/(V·s) |

*μ*_p_/*μ*_n_, carrier mobility; *ρ*, electrical resistivity; *p*/*n*, carrier concentration; *R*_H_, Hall coefficient

Table S3. Calculated formation energies of different defects in diamond.

| **Defects** | **Formation Energy (eV)** | **Defects** | **Formation Energy (eV)** |
| --- | --- | --- | --- |
| B- | 1.80 | B_4_N- | 2.53 |
| N- | 5.45 | BO- | 6.20 |
| O- | 10.31 | B_2_O- | 3.93 |
| BN- | 0.63 | B_3_O- | 3.47 |
| BN_2_- | 5.32 | B_4_O- | 3.25 |
| BN_3_- | 8.34 | BO_2_- | 14.96 |
| BN_4_- | 10.44 | BO_3_- | 22.42 |
| B_2_N- | 1.14 | BO_4_- | 33.00 |
| B_3_N- | 1.82 |  |  |

Table S4. Formation energies of hydrogen at various sites in BN_2_ co-doped diamond.

| **Site** | **Energy (eV)** | **Site** | **Energy (eV)** |
| --- | --- | --- | --- |
| Antibonding site of  the N-B bond | 28.50 | Bond-centered site of  the B-C bond | 29.82 |
| Bond-centered site of  the B-N bond | 28.32 | C-site adjacent to boron | 25.32 |
| C-site adjacent to nitrogen | 30.20 | Hexagonal site adjacent  to boron | 28.82 |
| Hexagonal site adjacent  to nitrogen | 28.51 | substitution site adjacent  to boron | 27.57 |
| substitution site adjacent  to nitrogen | 27.33 |  |  |

Table S5. Adsorption energies (eV) of nitrogen atoms at six high symmetry sites in diamond.

| **Structure** |  | **P1** | **P2** | **P3** | **P4** | **P5** | **P6** |
| --- | --- | --- | --- | --- | --- | --- | --- |
| No-H-D |  | 2.190 | 3.363 | 5.129 | 7.461 | 4.446 | 5.740 |
| All-H-D |  | 9.628 | 9.173 | 9.723 | 9.881 | 9.855 | 9.885 |
| 1H-D |  | 4.676 | 3.920 | 6.164 | 8.797 | 6.466 | 8.870 |
| 2H-D | DCC | 3.282 | 4.437 | 8.654 | 9.718 | 8.111 | 8.546 |
|  | DCO | 4.782 | 4.017 | 3.488 | 7.345 | 6.602 | 8.871 |
|  | DR | 5.100 | 4.366 | 5.692 | 7.588 | 3.438 | 7.570 |
| 3H-D | DC | 4.520 | 4.199 | 4.649 | 8.317 | 6.329 | 8.748 |
|  | DR | 5.033 | 4.329 | 7.185 | 7.397 | 3.048 | 5.827 |

Table S6. Adsorption energies (eV) of oxygen atoms at six high symmetry sites in diamond.

| **Structure** |  | **P1** | **P2** | **P3** | **P4** | **P5** | **P6** |
| --- | --- | --- | --- | --- | --- | --- | --- |
| No-H-D |  | 0.998 | 1.967 | 4.357 | 6.054 | 3.533 | 5.792 |
| All-H-D |  | 8.073 | 7.057 | 8.054 | 8.036 | 8.053 | 8.043 |
| 1H-D |  | 5.414 | 2.947 | 4.672 | 7.059 | 6.040 | 7.072 |
| 2H-D | DCC | 2.205 | 3.051 | 7.037 | 7.944 | 7.422 | 7.355 |
|  | DCO | 5.500 | 2.854 | 2.444 | 5.874 | 6.015 | 7.065 |
|  | DR | 5.730 | 2.783 | 4.788 | 6.191 | 2.293 | 6.302 |
| 3H-D | DC | 5.267 | 2.956 | 3.143 | 6.751 | 5.913 | 6.964 |
|  | DR | 5.644 | 2.736 | 4.705 | 6.089 | 2.261 | 6.220 |

Table S7. Adsorption energies (eV) of nitrogen at various high symmetry sites on different adsorption substrates.

|  | **P1** | **P2** | **P3** | **P4** | **P5** | **P6** |
| --- | --- | --- | --- | --- | --- | --- |
| Diamond | 2.190 | 3.363 | 5.129 | 7.462 | 4.446 | 5.740 |
| B-diamond | 1.624 | / | / | / | 3.832 | / |
| O-diamond | -0.493 | -0.489 | 3.333 | / | / | / |


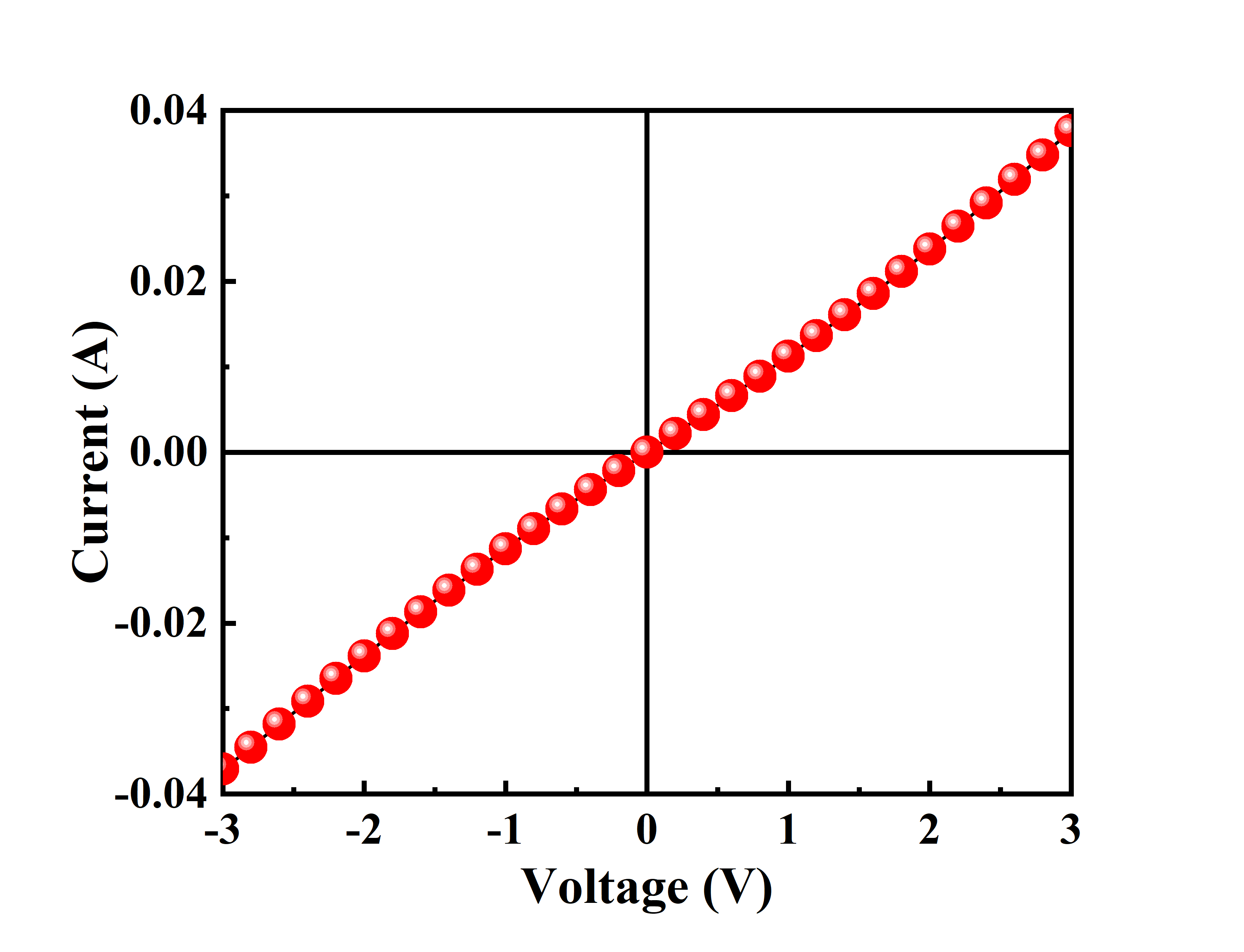


Figure S1. I-V characteristics of metal electrode and BNO-doped diamond.


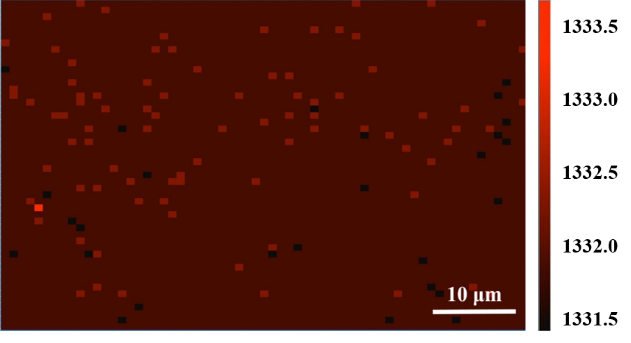

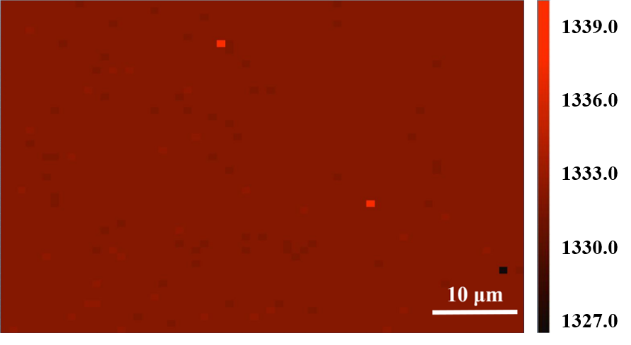


1. 990~1000 K


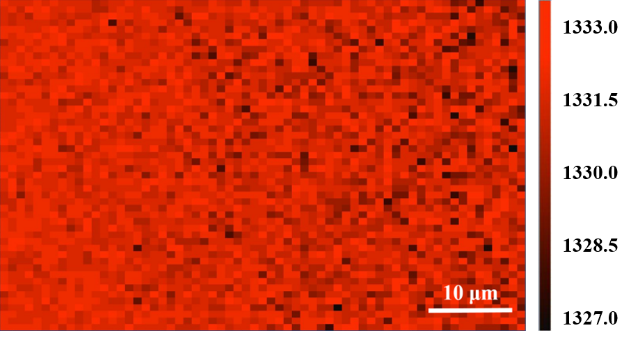

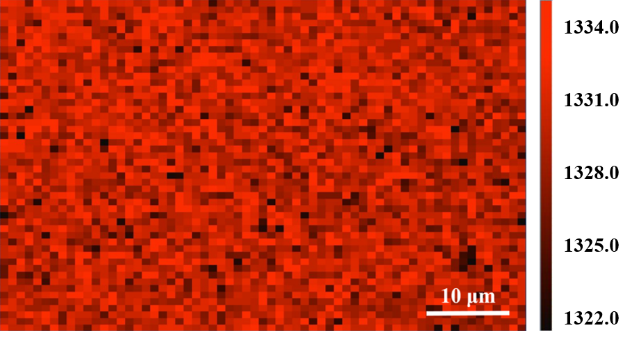


1. 1000~1010 K


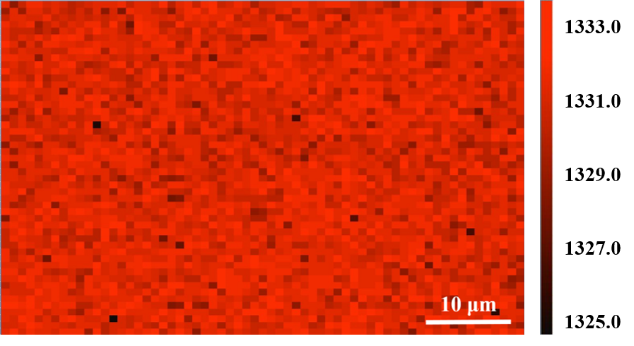

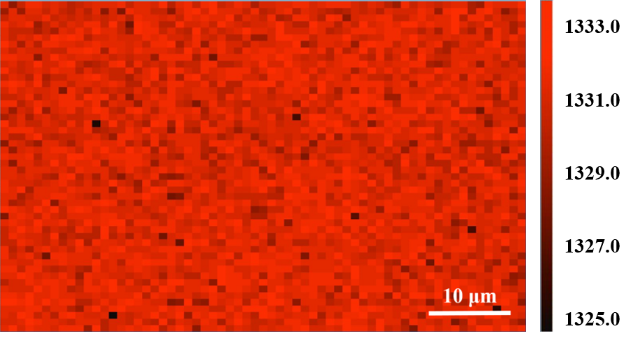


1. 1010~1020 K


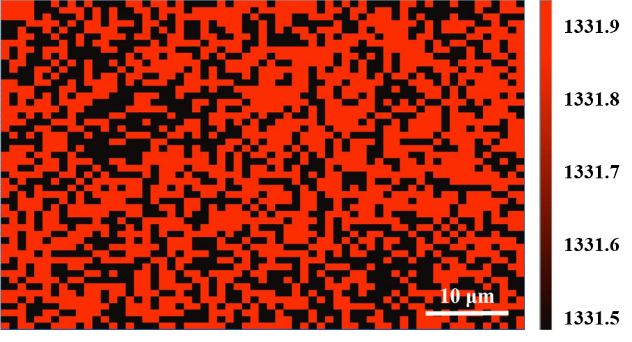

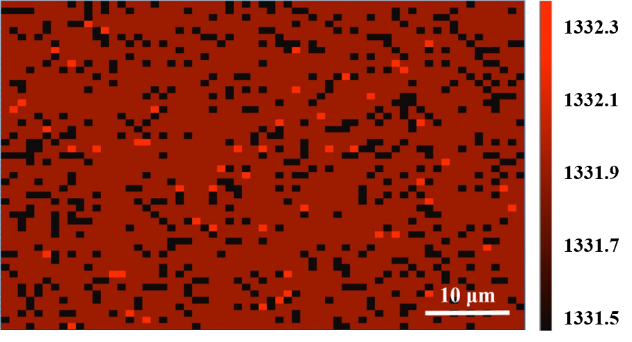


1. 1020~1030 K


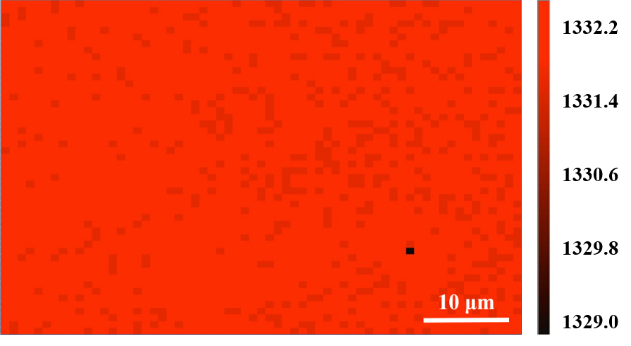

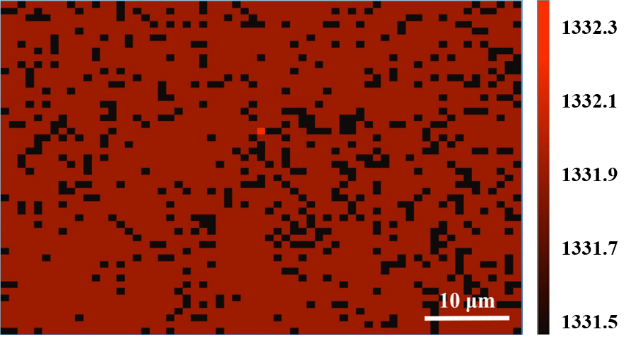


1. 1030~1040 K


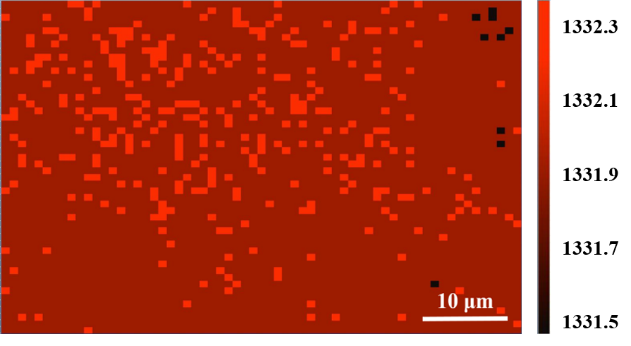

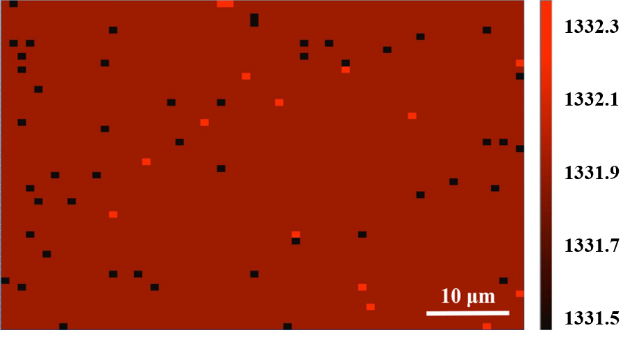


1. 1040~1050 K


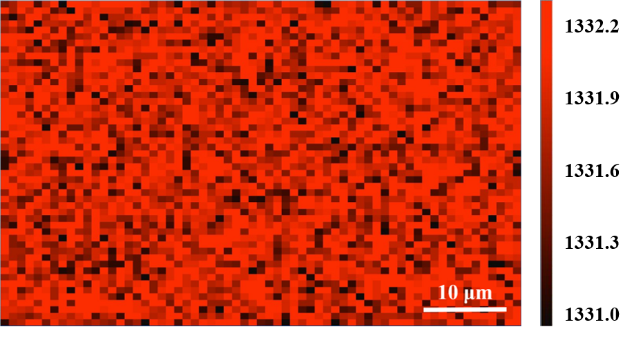

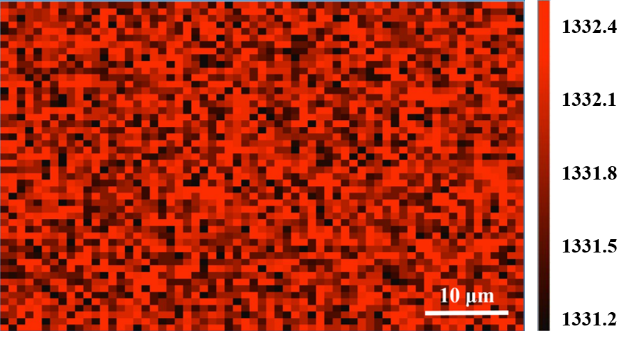


1. 1050~1060 K

Figure S2. Raman Mapping of the centre (left) and edge (right) regions of BNO co-doped diamond samples at different growth temperatures


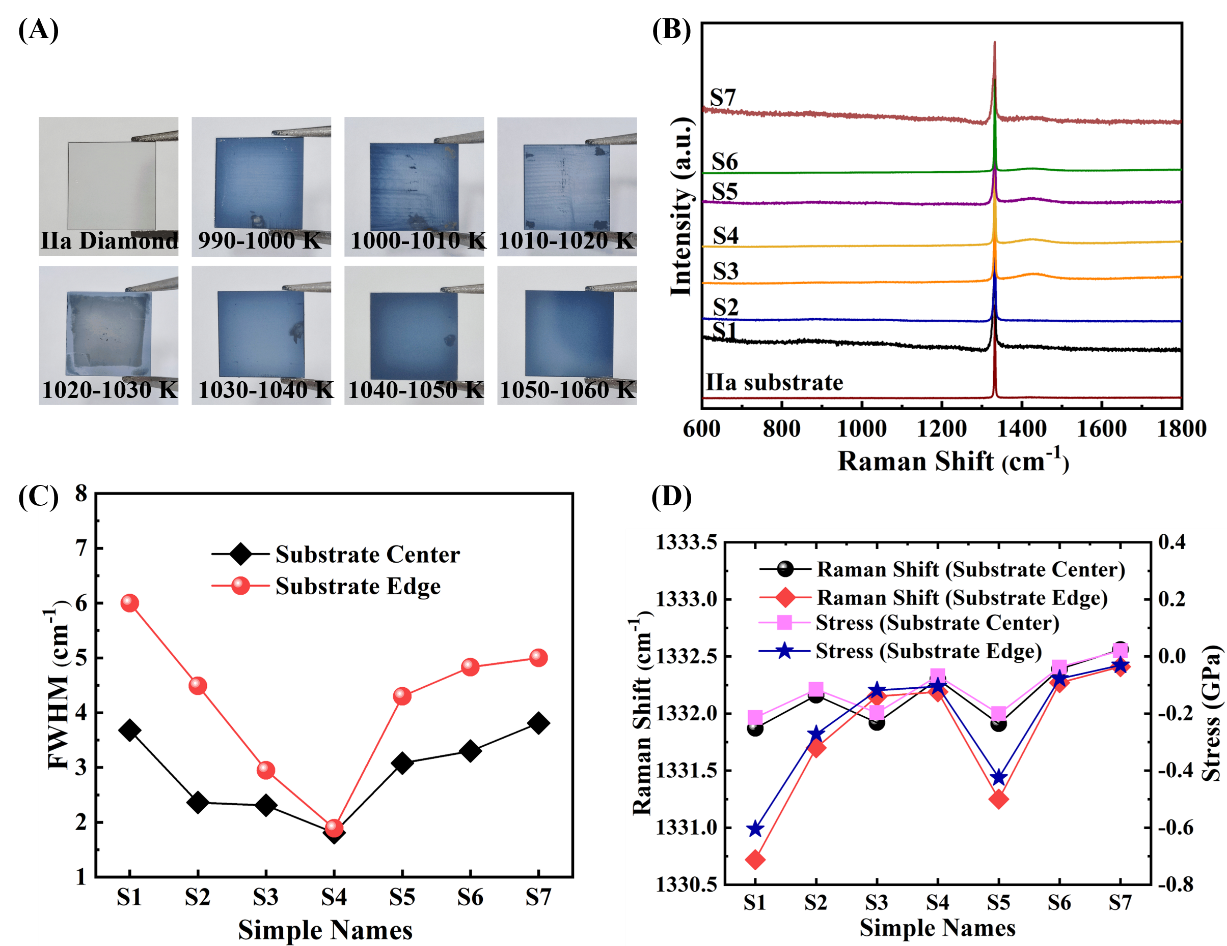


Figure S3. Optical images and Raman spectroscopy analysis of BNO co-doped diamond under different growth temperatures.


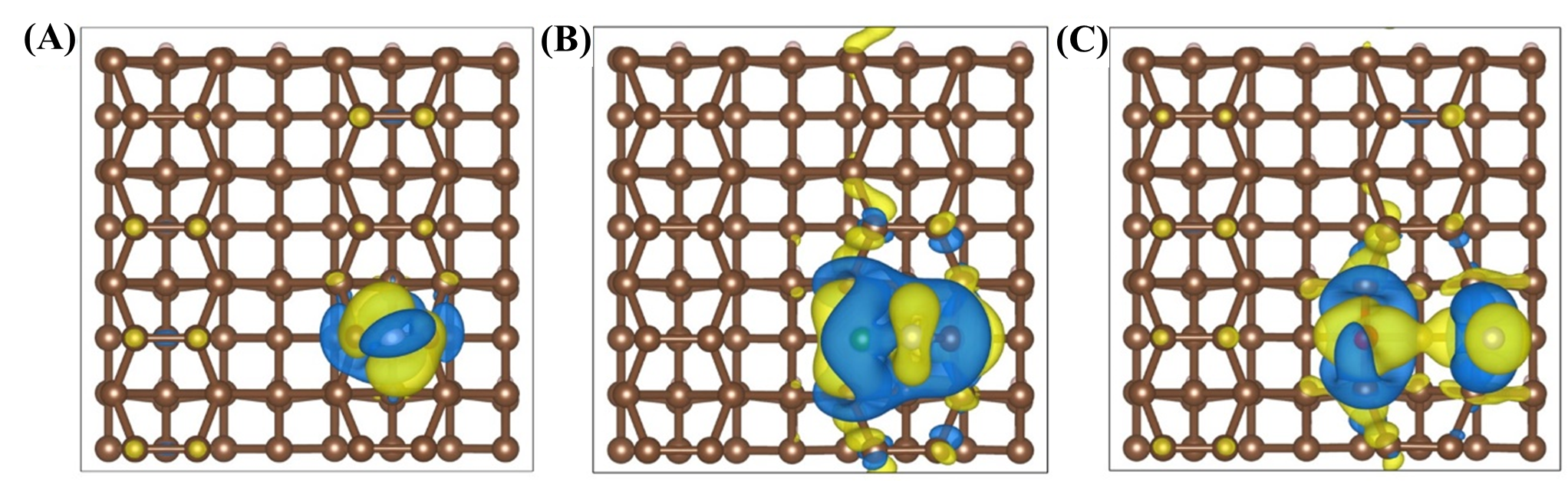


Figure S4. Differential charge density of nitrogen atoms on three different substrates. (a) Diamond; (b) boron-diamond; (c) oxygen-diamond.


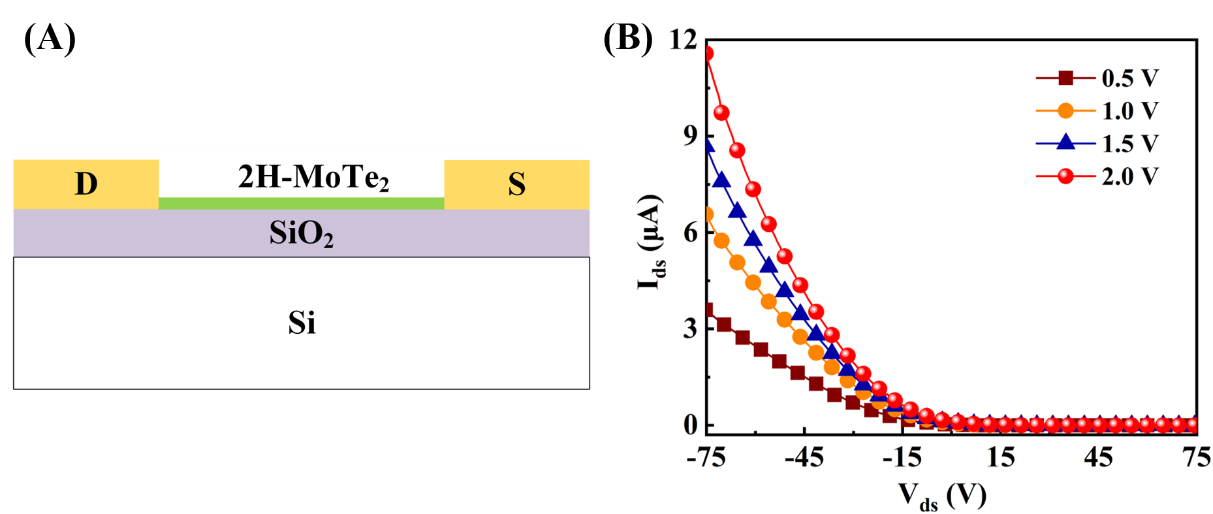


Figure S5. Electrical properties of 2H-MoTe_2_. (A) Structural schematic; (B) I-V characteristic curve.

**References**

[1] S. Koizumi, M. Kamo, Y. Sato, H. Ozaki, T. Inuzuka, Growth and characterization of phosphorous doped {111} homoepitaxial diamond thin films, Applied Physics Letters 71(8), 1065-1067 (1997).

[2] I. Sakaguchi, M. N.-Gamo, Y. Kikuchi, E. Yasu, H. Haneda, T. Suzuki, T. Ando, Sulfur: A donor dopant for n-type diamond semiconductors, Physical Review B 60(4), R2139-R2141 (1999).

[3] S. Koizumi, T. Teraji, H. Kanda, Phosphorus-doped chemical vapor deposition of diamond, Diamond and Related Materials 9(3), 935-940 (2000).

[4] Z. Teukam, J. Chevallier, C. Saguy, R. Kalish, D. Ballutaud, M. Barbé, F. Jomard, A. Tromson-Carli, C. Cytermann, J.E. Bulter, M. Bernard, C. Baron, A. Deneuville, Shallow donors with high n-type electrical conductivity in homoepitaxial deuterated boron-doped diamond layers, Nature materials 2(7), 482-486 (2003).

[5] M. Katagiri, J. Isoya, S. Koizumi, H. Kanda, Lightly phosphorus-doped homoepitaxial diamond films grown by chemical vapor deposition, Applied physics letters 85(26), 6365-6367 (2004).

[6] H. Kato, S. Yamasaki, H. Okushi, n-type doping of (001)-oriented single-crystalline diamond by phosphorus, Applied Physics Letters 86(22), (2005).

[7] H. Kato, T. Makino, S. Yamasaki, H. Okushi, n-type diamond growth by phosphorus doping on (0 0 1)-oriented surface, Journal of Physics D: Applied Physics 40(20), 6189 (2007).

[8] X.J. Hu, J.S. Ye, H. Hu, X.H. Chen, Y.G. Shen, Phosphorus ion implantation and annealing induced n-type conductivity and microstructure evolution in ultrananocrystalline diamond films, Applied Physics Letters 99(13), (2011).

[9] Y. Wang, Z. Yin, Structural and electrical properties of sulfur-doped diamond thin films, Plasma Science and Technology 16(3), 255 (2014).

[10] H. Kato, M. Ogura, T. Makino, D. Takeuchi, S. Yamasaki, N-type control of single-crystal diamond films by ultra-lightly phosphorus doping, Applied Physics Letters 109(14), (2016).

[11] H. Xu, H. Ye, D. Coathup, I.Z. Mitrovic, A.D. Weerakkody, X. Hu, An insight of p-type to n-type conductivity conversion in oxygen ion-implanted ultrananocrystalline diamond films by impedance spectroscopy, Applied Physics Letters 110(3), (2017).

[12] H. Zhang, S.S. Li, G.H. Li, T.C. Su, M.H. Hu, H.A. Ma, X.P. Jia, Y. Li, Effect of B-S co-doping on large diamonds synthesis under high pressure and high temperature, International Journal of Refractory Metals and Hard Materials 66, 26-30 (2017).

[13] X.B. Liu, X. Chen, D.J. Singh, R.A. Stern, J.S. Wu, S. Petitgirard, C.R. Bina, S.D. Jacobsen, Boron–oxygen complex yields n-type surface layer in semiconducting diamond, Proceedings of the National Academy of Sciences 116(16), 7703-7711 (2019).

[14] I. Stenger, M.A. Pinault-Thaury, N. Temahuki, R. Gillet, S. Temgoua, H. Bensalah, E. Chikoidze, Y. Dumont, J.Barjon, Electron mobility in (100) homoepitaxial layers of phosphorus-doped diamond, Journal of Applied Physics 129(10), (2021).

[15] M.A. Pinault-Thaury, I. Stenger, R. Gillet, S. Temgoua, E. Chikoidze, Y. Dumont, F. Jomard, T. Kociniewski, J. Barjon, Attractive electron mobility in (113) n-type phosphorus-doped homoepitaxial diamond, Carbon 175, 254-258 (2021).

[16] D.Y. Liu, L.C. Hao, Y. Teng, F. Qin, Y. Shen, K. Tang, J.D. Ye, S.M. Zhu, R. Zhang, Y.D. Zheng, S.L Ge, Nitrogen modulation of boron doping behavior for accessible n-type diamond, APL Materials 9(8), (2021).

[17] C. Chen, D. Fan, H. Xu, M. Jiang, X. Li, S. Lu, C. Ke, X. Hu, Monoatomic tantalum induces ordinary-pressure phase transition from graphite to n-type diamond, Carbon 196, 466-473 (2022).

[18] W. Jia, Z. Cao, L. Wang, J. Fu, X. Chi, W. Gao, L. Wang, The analysis of a plane wave pseudopotential density functional theory code on a GPU machine, Computer Physics Communications 184(1), 9-18 (2013).

[19] J.P. Perdew, K. Burke, M. Ernzerhof, Generalized gradient approximation made simple, Physical review letters 77(18), 3865 (1996).

[20] D. Hamann, Optimized norm-conserving Vanderbilt pseudopotentials, Physical Review B 88(8), 085117 (2013).

[21] H.J. Monkhorst, J.D. Pack, Special points for Brillouin-zone integrations, Physical review B 13(12), 5188 (1976).

[22] T. Hom, W. Kiszenik, B. Post, Accurate lattice constants from multiple reflection measurements. II. Lattice constants of germanium silicon, and diamond, Journal of Applied Crystallography 8(4), 457-458 (1975).

[23] Z.J. Suo, J.W. Luo, S.S. Li, L.W. Wang, Image charge interaction correction in charged-defect calculations, Physical Review B 102(17), 174110 (2020).

[24] C. Freysoldt, B. Grabowski, T. Hickel, J. Neugebauer, G. Kresse, A. Janotti, C.G. Van De Walle, First-principles calculations for point defects in solids, Reviews of Modern Physics 86(1), 253 (2014).
